# Echoes of L1 Syllable Structure in L2 Phoneme Recognition

**DOI:** 10.3389/fpsyg.2021.515237

**Published:** 2021-07-20

**Authors:** Kanako Yasufuku, Gabriel Doyle

**Affiliations:** Department of Linguistics and Asian/Middle Eastern Languages, San Diego State University, San Diego, CA, United States

**Keywords:** phonetics, phonology, phonotactics, language acquisition, McGurk effect, cue combination, syllables

## Abstract

Learning to move from auditory signals to phonemic categories is a crucial component of first, second, and multilingual language acquisition. In L1 and simultaneous multilingual acquisition, learners build up phonological knowledge to structure their perception within a language. For sequential multilinguals, this knowledge may support or interfere with acquiring language-specific representations for a new phonemic categorization system. Syllable structure is a part of this phonological knowledge, and language-specific syllabification preferences influence language acquisition, including early word segmentation. As a result, we expect to see language-specific syllable structure influencing speech perception as well. Initial evidence of an effect appears in Ali et al. (2011), who argued that cross-linguistic differences in McGurk fusion within a syllable reflected listeners’ language-specific syllabification preferences. Building on a framework from Cho and McQueen (2006), we argue that this could reflect the Phonological-Superiority Hypothesis (differences in L1 syllabification preferences make some syllabic positions harder to classify than others) or the Phonetic-Superiority Hypothesis (the acoustic qualities of speech sounds in some positions make it difficult to perceive unfamiliar sounds). However, their design does not distinguish between these two hypotheses. The current research study extends the work of Ali et al. (2011) by testing Japanese, and adding audio-only and congruent audio-visual stimuli to test the effects of syllabification preferences beyond just McGurk fusion. Eighteen native English speakers and 18 native Japanese speakers were asked to transcribe nonsense words in an artificial language. English allows stop consonants in syllable codas while Japanese heavily restricts them, but both groups showed similar patterns of McGurk fusion in stop codas. This is inconsistent with the Phonological-Superiority Hypothesis. However, when visual information was added, the phonetic influences on transcription accuracy largely disappeared. This is inconsistent with the Phonetic-Superiority Hypothesis. We argue from these results that neither acoustic informativity nor interference of a listener’s phonological knowledge is superior, and sketch a cognitively inspired rational cue integration framework as a third hypothesis to explain how L1 phonological knowledge affects L2 perception.

## Introduction

Second language acquisition and representation is extensively affected by a learner’s knowledge of other languages. These effects appear at many levels, including lexical (Schulpen et al., 2003; Weber and Cutler, 2004; Otake, 2007) and phonological recognition (Dupoux et al., 1999; Carlson et al., 2016). This can happen even at an abstract level, with first language (L1) knowledge helping second/later language (L2) learners identify which phonetic dimensions are used for phonemic discrimination in the new language (Lin, 2001; Pajak and Levy, 2012). In this paper, we examine how difficulties in L2 phonemic categorization may arise from the difference between the L1 and L2 phonological structure (in the form of syllabification preferences) and the quality of the acoustic/visual input. We find evidence that both phonological and phonetic cues are used by L2 learners to identify sound categories, and that the learners’ use of these cues varies depending on the input they are given and their L1 experience. This argues against previous proposals, Phonetic- and Phonological-Superiority Hypotheses, that claim one cue consistently outweighs the other (Cho and McQueen, 2006; Ali et al., 2011), and suggests that learners may be capable of combining information from their L1 structure with perceived cue-reliability as part of a rational cue integration framework for L2 perception and categorization.

Whether we consider first or later language acquisition, learning to effectively represent the acoustic signals available in language input and building these observations up into phonological structure for the target language are key components of language learning. L1 learners build their fundamental understanding of language-specific sound patterns by analyzing acoustic data in the input and applying this knowledge in later speech perception and language comprehension (Maye et al., 2002; Werker et al., 2007). L2 learners follow a similar process, but may also be influenced by the structure they have already developed from their L1. In some cases, this may be helpful; if a learner’s L1 has the same structure as the target L2, the L1 can provide a head-start for learning the target L2 structure (Best and Tyler, 2007; Pajak and Levy, 2012). If, on the other hand, the L1 has a different structure, the L1 could impede L2 acquisition. This second case is a common pitfall for language learners anecdotally, and has been backed up by research showing that learners encounter more difficulty perceiving and processing linguistic features that are present in the L2 but either absent or significantly different in their L1 (Best and Tyler, 2007). This difficulty may be due to the linguistic knowledge of the languages previously acquired, which L2 learners activate during the course of language processing and comprehension (Dupoux et al., 1999; Carlson et al., 2016).

L1 knowledge can influence perceived L2 phonetic and phonological structure in many ways. At perhaps the most basic level, L2 phonemic distinctions that are present in a learner’s L1 can be easier to perceive than those that are not present or merely allophonic in the L1 (Flege, 2003). For instance, Spanish-Catalan bilinguals whose L1 is Spanish find it more difficult to distinguish word pairs that only differ in one phoneme (i.e., /e/ vs. /ε/) than Catalan-dominant Catalan-Spanish bilinguals (Palliar et al., 2001). This reflects the influence of Catalan phonology, since both /e/ and /ε/ are phonemically contrastive in Catalan while only /e/ exists in Spanish phonemic inventory. In addition, distinctions that are present in both L1 and L2, but with different boundaries in the two languages, also show an effect of L1. L2 learners tend to draw category boundaries between ambiguous sounds that are in line with the acoustic boundaries of their native language (Escudero and Vasiliev, 2011; Kartushina and Frauenfelder, 2013).

These effects extend beyond the L1 phonemic inventory and boundaries as well. Higher-level phonological knowledge from L1, such as syllabic structure and phonotactic restrictions, can also contribute to inaccuracies in perceiving or representing L2 speech sounds. L2 learners often report illusory segments in L2 perception that bring the L2 observation closer to L1 phonotactics. Carlson et al. (2016) asked L1 Spanish-speakers to listen to L2 words with an initial consonant cluster composed of /s/ followed by another consonant (i.e., #sC_). Such initial clusters are phonotactically illicit in Spanish, and the participants reported hearing an illusory vowel /e/ before the word-initial consonant cluster #sC, which would be acceptable in Spanish phonotactics. Dupoux et al. (1999) found that Japanese listeners reported hearing an illusory vowel [u] between consonants when they were presented to VCCV as well as VCuCV nonwords while French listeners, whose L1 allows to have a consonant cluster, were able to judge when the vowel was absent. A likely explanation for this epenthetic repair pattern is that an illusory vowel was inserted after the final consonants to break the VC syllable into a combination of V followed by CV syllables, fitting the standard Japanese syllable structure, (C)V, and avoid a phonotactic violation. Indeed, this epenthetic repair has also been observed in Japanese loanword adaptation (insert /i/ when word is ending with a [tʃ] and [dʒ], /o/ after [t] and [d], and /ɯ/ in other phonetic environment).

A similar example comes from English listeners, who miscategorize certain onset phonemes in a way that fits better with English phonotactics. English-speaking listeners exposed to onset consonant clusters composed of a fricative consonant (e.g., [ʃ] and [s]) followed by a liquid consonant (e.g., [ɹ ] and [l]) tend to report hearing [ʃɹi] and [sli], compared to the phonotactically worse (within English) [sɹi] and [ʃli], even though the same fricative was being played. That is, even though the actual sounds were acoustically close to [ʃ], when it appears in the phonetic context [_li], English speakers reported that they heard [sli] rather than [ʃli]. In contrast, when an [s]-like sound was played in a phonetic context such as [_ɹ i], English speakers perceived it as [ʃɹ i] rather than [sɹ i] (Massaro and Cohen, 2000). This supports the idea that speech perception is heavily influenced by listeners’ L1 phonotactic knowledge, which includes detailed phonotactic information and syllable structure preferences. Most importantly, it shows that effective acquisition of the phonemic inventory of an L2 is not just a matter of learning the new language’s phonemic inventory and phonetic boundaries, but also a matter of learning the language’s abstract structure, including phonotactics, to produce accurate sound categorization.

Building on this idea of abstract L1 influences, some studies have revealed that listeners of L1s with different syllable structure preferences syllabify unfamiliar strings of sounds differently, in line with the predominant syllable structure of their L1 (Cutler et al., 1986; Otake et al., 1993). These differences have often been tested by asking listeners to syllabify words in their L1 or an L2, but this does not directly get at the question of whether the syllabification biases from L1 have any effects on perception itself.

An alternative way of testing this that directly measures perception relies on the McGurk effect, in which observers are played audio and video information that do not agree, and report hearing a sound that is inconsistent with the audio information. For instance, a common cross-linguistic McGurk “fusion” effect is perceiving a [t] sound when presented with audio of a [p] sound but video of a [k] sound (McGurk and MacDonald, 1976; Tiippana et al., 2004). Researchers have examined cross-linguistic differences in English and Arabic speakers’ perception of monosyllabic CVC words, using McGurk fusion rate differences to argue for an influence of L1 syllable structure preferences on sound perception in onset and coda positions (Ali and Ingleby, 2002; Ali, 2003; Ali and Ingleby, 2005). The results show that English-speaking participants fused audio-visually incongruent English consonant recordings more when they were in coda position than onset position, while Arabic speakers did not show such positional differences on similar stimuli in Arabic. Based on these L1 results, they argued that, despite both languages allowing surface-form coda consonants, the abstract representation of Arabic coda consonants is as an onset in a syllable with an illusory vowel (similar to Dupoux et al., 1999 and Carlson et al., 2016’s findings).

To argue that this difference is the result of syllable representation and a preference for certain syllable structure, Ali et al. (2011) further tested English learners of Arabic (with an average of 3 years of part-time Arabic instruction) in addition to English and Arabic monolinguals. English speakers with significant Arabic instruction showed a similar response to Arabic monolinguals when they were presented with Arabic stimuli: they fused audio and visual information almost equally in onset and coda positions. English speakers without Arabic exposure showed a similar response on both English and Arabic stimuli: increased fusion in codas versus onsets. Based on these results, they argued that an L1 syllabic structure effect was present, that English and Arabic had different mental representations of syllabic structure, and that the L1 effect could be overcome with sufficient exposure to the L2. This suggests that high-level phonological structure from L1 can introduce significant difficulties for L2 perception and categorization. However, there are at least two possible explanations for this data.

The first explanation is that, as Ali et al. (2011) claimed, the difference in the magnitude of McGurk fusion rate is dependent on the phonological syllabic structure, and thus reflects language-specific phonological preferences inherited from L1. This supports the Phonological-Superiority Hypothesis, which claims that cross-linguistic differences in consonant perception in different syllable positions arise from differences in the native phonological structure preferences (Cho and McQueen, 2006). When learners encounter L2 speech, especially when sounds appear in unfamiliar sequences or phonotactically illicit positions according to the L1, they re-analyze it to fit an L1-acceptable syllable structure rather than maintaining the more accurate L2 structure. Ali et al. (2011) argue that English monolinguals show more fusion on Arabic codas than Arabic speakers do because they represent the Arabic coda (which does not show increased fusion) as if it were an English coda (which does show increased fusion). Given the findings that listeners can re-interpret L2 sounds to fit their L1 (as with illusory epenthetic vowels of Dupoux et al. (1999) and Carlson et al. (2016)), such differences in representation are reasonable, and could induce some phonological effects on perception. However, Ali et al. (2011) do not propose a mechanism to explain how abstract differences in phonological structure representations (as an onset versus a coda) create strong perceptual differences in audio-visual integration.

An alternative, more explicitly mechanistic, approach proposes that the perceptual differences across syllabic positions come from differences in the actual phonetic realizations of the sounds in different positions. The English coda consonants tested in the previous studies may be acoustically less informative compared to onset consonants; English speakers do not have to audibly release word-final stops, and unreleased stops are harder to identify (Lisker, 1999). In addition, although we use the same phonetic symbol to represent a sound category in onset and coda positions, the acoustic signal for onset and coda versions of a phone can be very different. This is especially true for stops, where the acoustically informative transition between vowel and closure changes occurs at the beginning of a coda stop but at the end of an onset stop. Recognizing the representational equivalence of the acoustically distinct onset and coda forms of the same L2 phoneme may not be trivial to the learner (Tsukada, 2004). This would fit the Phonetic-Superiority Hypothesis, which argues that difficulties arise from differences in the quality of the phonetic identifiability of some sounds, independent of the phonological system of the speaker’s native language (Cho and McQueen, 2006).

Previous work has found cases where L2 perception is relatively independent of L1 phonotactic restrictions, suggesting that phonetic differences can be perceptually salient despite cross-linguistic phonological differences. For instance, Dutch speakers are able to perceive and discriminate voicing differences in English word-final fricatives (i.e., /s/-/z/ and /f/-/v/ contrasts) and stops (i.e., /p/-/b/ and /t/-/d/ contrasts) as accurately as in word-initial position, even though Dutch neutralizes voicing contrasts in word-final position (Broersma, 2005). Similarly, native Japanese speakers are able to discriminate English /r/ and /l/ more accurately in syllable-final position than in other positions, despite this not representing a phonemic distinction in any position in Japanese (Sheldon and Strange, 1982). This seems to argue against the Phonological-Superiority Hypothesis, since Japanese is primarily a CV syllable language, yet the best performance was occurring in a phonotactically illicit position. Sheldon and Strange attributed the finding to the actual acoustic difference between English /r/ and /l/ sounds, which may be more acoustically distinct in word-final position than word-initial position, supporting the dominance of phonetic over phonological perceptual influences. Especially in the case of the audio-visual integration of Ali et al. (2011), acoustically less informative audio input may have caused English and Arabic listeners to pay more attention to the visually presented information in English coda productions, increasing McGurk fusion rates because of less phonetic clarity. Because languages differ in their phonemic inventories and divisions, difficulties that appear to depend on the L1 structure may actually stem primarily from the joint difficulties of the task and the individual phone productions.

Some research studies have carefully investigated the interaction of phonetic and phonological information when distinguishing phoneme contrasts in different phonotactic contexts. Tsukada (2004) investigated how speakers of different languages discriminate Thai and English voiceless stop contrasts (i.e., /p/-/t/, /p/-/k/, and /t/-/k/) in syllable-final positions. English and Thai both have three-way stop contrasts in syllable-final position, but English final stops can be released or unreleased, while Thai final stops are obligatorily unreleased. Tsukada played recordings of English (released) and Thai (unreleased) CVC words to both English and Thai listeners. English and Thai listeners showed equal accuracy in discriminating English speakers’ stop contrasts, but Thai listeners were more accurate than English listeners in discriminating Thai speakers’ contrasts. Tsukada argued that the asymmetry may be due to the difference in acoustic information available in the Thai case, since unreleased stops are acoustically less informative. Tsukada and Ishihara (2007) followed up on this idea by exposing Japanese listeners to word-final English and Thai stop contrasts. Japanese has the same voiceless stop inventory as Thai and English, but Japanese phonotactics reject stops in coda position. Their results show that all three language groups correctly discriminate English word-final stop contrasts, but for unreleased Thai-language recordings, Thai listeners were more accurate than English listeners who, in turn, were more accurate than Japanese listeners.

These results suggest that the difficulties in perceiving L2 speech sounds may be motivated by the listeners’ L1 structure biases, but the degree of difficulty may vary depending on the actual acoustic cue informativity. In the present study, in addition to investigating how phonetic information and phonological information would influence L2 speech recognition, we would like to address the question of how listeners balance their reliance on phonetic and phonological factors when categorizing L2 phonemes. Phonetic and phonological influences on L2 phoneme categorizations suggest two different potential problems facing the L2 learner, one general and one specific to the L1. On the phonetic side, L2 learners may have to deal with a language that provides data of differing acoustic quality, making certain contrasts or phonotactic positions difficult to classify accurately. Learners then would have to learn to extract the information that they need to categorize sounds correctly. On the phonological side, L2 learners may need to learn to represent the phonological and syllabic structure of the L2 (if their L1 uses a different representation) to properly identify the phones. In the present work, we will look for signs of each of these problems in L2 representation. We will test to see if one difficulty is stronger than the other, as the Phonetic- and Phonological-Superiority Hypotheses propose, or if the two have a more complex relationship.

To see how such factors may interact in L1 and L2 perception, consider the possible explanations that each hypothesis offers for the findings of Ali et al. (2011) that English listeners show increased McGurk fusion (i.e., decreased ability to identify the correct phoneme from acoustic information) in codas versus onsets, while Arabic speakers (even advanced-L2 Arabic speakers) show no such difference. At first, this may seem to be clear-cut evidence of phonological superiority. The English speakers phonologically represent the stop consonants as onsets and codas, and categorize them differently. The Arabic speakers might represent onsets and codas identically, and thus show no difference in categorization accuracy. However, as mentioned above, this presupposes an unstated mechanism to explain why codas would be inherently inclined toward less accurate representation than onsets. How could phonetic superiority explain this? Suppose onsets are more acoustically identifiable than codas in general. English speakers’ accuracies are as expected, with worse accuracy on codas. Arabic speakers’ accuracies require an explanation, since they seem unaffected by this acoustic difference. Here we encounter two confounds in Ali et al.’s experimental design: the Arabic stimuli were real Arabic words, and Arabic speakers were only tested on Arabic, not English. Since Arabic speakers knew the potential words they were trying to identify, they may have been able to rely on prior knowledge about the wordforms to convert this to a lexical recognition task instead of a phonemic categorization task. Without Arabic listeners’ data on English wordforms, it is difficult to identify a specific mechanism for the differences, and each relies in part on supposition.

To fill these gaps and more directly test the relative influences of phonetic and phonological representation on phoneme categorization, we propose a study that examines L1 effects on listeners’ representation of an artificial language. This removes the potential lexical recognition confound and equalizes the listeners’ inherent familiarity with the test words. We also independently assess listeners’ accuracy on audio-only, audio-visual congruent, and audio-visual incongruent tasks. This builds a baseline to understand if the proposed onset-coda differences in acoustic informativity are real, and if they differ due to previous language exposure. We extend to congruent and incongruent audio-visual data to test both how L1 knowledge may affect L2 phonemic categorization in real-world applications, and how the phonetic and phonological structures interact.

We compare L1 English and Japanese speakers, taking advantage of the different underlying phonological representations of syllable they have. Whereas both Arabic and English allow stop consonants in the surface coda position (requiring Ali et al., 2011, to consider potential differences in the abstract syllable structure), Japanese blocks stop consonants from almost all coda positions at the surface, presenting a more robust phonotactic difference. Japanese and English are ideal languages to address the current research questions because they are languages with substantially different phonologically defined syllables. Japanese, aside from limited exceptions for nasals and geminates, disallows coda consonant clusters. The complete syllable inventory of Japanese consists of V, VV, CV, CVV, CVN (i.e., consonant-vowel-nasal), and CVQ (i.e., consonant-vowel-first half of a geminate consonant, extending into a following syllable). In contrast, English has relatively flexible syllable structures and allows consonant clusters consisting of a wide range of onset or coda consonants (including CCCVCCC, as in *strengths*). This makes Japanese a good comparison language for examining how the language-specific syllable structure preferences influence phonetic perception, and whether the perception of audio-visual incongruent sound is influenced primarily by listeners’ L1 phonological knowledge, the actual acoustic information available in the test items, or some other linguistic factor such as lexical knowledge. Using a set of nonsense monosyllabic CVC words and introducing them to participants as a novel language limits participants’ ability to rely on any other factors that may influence speech perception.

Consequently, we would like to address two research questions in the current study: (1) Do we see the signs of both phonetic and phonological influence in audio-visual information integration during speech perception, or does one dominate the other, as the Phonetic- and Phonological-Superiority Hypotheses predict? (2) If we see an interaction of phonetic and phonological cues, what causes the shift between phonetic and phonological preferences in speech perception? If Japanese listeners demonstrate relatively less accuracy in detecting coda consonants compared to onset consonants in audio-only condition while English listeners perceive consonants in both positions accurately in the same condition, and Japanese listeners’ response patterns to audio-visual incongruent stimuli differ from those of English listeners’, this will be evidence for the Phonological-Superiority Hypothesis, since L1 structures drive the performance differences. In contrast, if the Japanese and English listeners have smaller differences due to linguistic influence than due to the specific stimuli they are classifying, this will be evidence for Phonetic-Superiority Hypothesis, since differences in phonetic clarity drive the performance differences. Finally, if the response patterns show L1-dependent differences in some stimuli but not others, this may be evidence for a third possibility: that both phonetic information and phonological knowledge influence speech perception, and their relative importance may vary depending on the informativity and reliability of each cue available during the course of speech perception, rather than one dominating the other overall.

## Methodology

### Participants

Twenty native English speakers (9 male, 11 female, age range of 11–44 years old, and mean age = 23.1 years old) and 20 native Japanese speakers (6 male, 14 females, age range of 19-53, and mean age = 22.1 years old) were recruited from San Diego State University and the associated American Language Institute (ALI). Data from two English-speaking participants were excluded from the data analysis due to their failure to understand the task. Data from two Japanese-speaking participants were excluded from the data analysis due to significant self-reported L3 exposure. None of the participants in either language group had lived in a foreign country for longer than a year, and all self-reported that they have normal hearing and vision. Averages of self-reported language proficiency level in their native language and second language(s) are provided in Table 1.

**TABLE 1:** Participants’ Self-Reported Language Proficiency in their native language, their second best language, and any third language experience.

	English participants		
	Language #1 (English)	Language #2	Language #3(if applicable)
Speaking	9.4	4.35	3.4
Understanding	9.55	4.75	3.91
Reading	9.65	4.7	3.9

	**Japanese participants**		
	**Language #1 (Japanese)**	**Language #2**	**Language #3(if applicable)**

Speaking	9.1	4.35	1.93
Understanding	8.95	4.9	2.07
Reading	8.45	4.85	2.47

*Values reported on a 10-point scale, with 1 being the lowest and 10 being the highest.*

The Japanese speakers had studied English as a second language at a Japanese educational institution for at least 3 years, as it is common for Japanese middle schools to require English as a foreign language class. Averages of self-reported English language proficiency levels in native Japanese speakers were 4.53 for speaking, 5.18 for understanding, and 5.06 for writing on a 10-point scale. There was only one English participant who had studied Japanese at college. This participant was kept in the analysis, as his overall self-reported language proficiency level in Japanese was only 3 of 10. The additional languages reported by Japanese speakers were Korean, Mandarin Chinese, Spanish, French, an unspecified Nigerian language, and Arabic and those reported by English speakers were Vietnamese, Spanish, French, Korean, American Sign Language, Mandarin Chinese, German, Gujurati, and Japanese. Four participants whose L1 is English reported that they had some exposure to a third language in childhood. They reported that English is now their dominant language, and their childhood language exposure was to languages that allow the coda consonants being used in the current study, and thus, their phonotactics should be similar to monolingual English speakers within the context of this experiment. As such, data from those participants were included in the analysis.

### Stimuli

To minimize the influence of word frequency, word recognition effects, and familiarity, and to control the lexical and phonetic information that could possibly influence the phonemic recognition and McGurk fusion effect, we created six monosyllabic (i.e., CVC) and six bisyllabic (i.e., CVCV) nonsense words in the current study. A complete list of nonsense words used as target stimuli can be found in Table 2.

**TABLE 2:** Target stimuli, in IPA (Monosyllabic CVC words and Bisyllabic CVCV words, which were treated as additional fillers in the current data analysis).

CVC words (targets)		CVCV words (fillers)	
pεm	zop	pεza	wopa
tεm	zot	tεza	wota
kεm	zok	kεza	woka

To focus on investigating the main factors influencing the difference in McGurk fusion rate depending on the consonant positions, the CVCV words were used as extra fillers in addition to 14 monosyllabic and 14 bisyllabic filler words. All the words were phonemically and phonotactically valid in both English and Japanese, except for the coda consonants in the CVC words in Japanese. A linguistically trained female native English speaker was video-recorded by a video camera (Panasonic 4K PROFESSIONAL) and audio-recorded by a separate microphone (SHURE KSM44A) while pronouncing these nonsense words transcribed using IPA. Each word was pronounced three times by the talker. We selected the secondly pronounced word as target stimulus to avoid list-final intonation. The video recordings were done in a soundproof booth at San Diego State University. Each word’s recording was cropped to be approximately 1500 ms.

For the audio-only stimuli, the audio file of each video was extracted from video recordings and stored as a WAV file. For the audio-video congruent files, the video kept its original audio and was exported to a Quicktime MOV file. For incongruent audio-visual pairs, each CVC stimulus containing audio /p/ was paired with the corresponding visual /t/ and /k/, and likewise, audio /k/ with visual /p/ and /t/, resulting in eight monosyllabic incongruent audio-visual stimuli. The audio was dubbed on video by matching the onset of the target consonant in the audio file and the video within 20 ms. As a result, a total of eight audio-visually incongruent CVC words and six audio-visually congruent CVC words were created as test stimuli in addition to 42 filler words (8 audio-visually incongruent bisyllabic words, 6 audio-visually congruent words, 14 monosyllabic congruent filler words, and 14 bisyllabic congruent filler words). All the stimulus editing was done by Adobe Premiere Pro CC software.

To discourage participants from attending exclusively to audio information, noise was added to the audio recordings. Multi-talker babble (MTB) was used as background noise to mimic a situation where participants listening to a conversation in a busy café or public space. Since listeners with normal hearing can be more adversely affected by multi-talker babble in their native language than babble in other languages (Van Engen and Bradlow, 2007), we created the MTB with recordings of both Japanese-native and English-native speakers. The MTB consisted of three “stories” created by randomly sequencing the filler words used in the experimental phase. Each story consisted of three to four nonsense sentences and is approximately 20 s long. Three native English speakers (1 male and 2 female) and 3 native Japanese speakers (1 male and 2 female) were recorded for each version. All six recordings were combined and loudness-normalized using Audacity 2.3.0.

### Procedure

The experimental session was conducted in the participant’s native language, either in English or Japanese. After filling out the consent form and language background questionnaire, each participant took four practice trials, consisting of two congruent audio-visual filler bisyllabic words and two audio-only filler bisyllabic words, so that participants would understand both types of stimuli in the experiment. The first instance of each was presented without MTB, so that the participant could recognize the voice of the main speaker apart from the background noise. After completing the practice trials, the investigator confirmed that the participant understood the experiment and then participants were left to complete the experiment alone, in a closed, quiet room.

The experiment consisted of two blocks: an audio-only block and an audio-visual block. The order of these blocks was counterbalanced among participants. In the audio-only block, each participant listened to a total of 40 words: 6 monosyllabic targets, 14 monosyllabic fillers, and 20 bisyllabic fillers. In the audio-visual block, each participant watched 52 words: 14 monosyllabic targets, 14 monosyllabic fillers, and 28 bisyllabic fillers. The order of the stimuli was randomized within each block. In both blocks, static pictures of the speaker were shown for 1500 ms before the target recording played. In the audio-only block, the static picture of the speaker continued to be shown while the target audio played, while in the audio-visual block, the video played at the same time as the audio. After the target audio, a different static picture of the speaker appeared for another 1000 ms. MTB played at a constant volume throughout all three segments. After the 1000 ms static picture, the MTB stopped, and text on the screen asked participants to type in the word that they thought they heard. The next trial started whenever participants hit the space key, so that participants could take breaks as needed, and there was no feedback given during the experiment.

Each participant was asked to listen to a series of words in an unfamiliar language played through Labsonic LS255 School Headphones while watching either a video or a static picture of the main speaker on the 13″ screen of a MacBook Air laptop. The laptop was placed on a standing desk riser Tao Tronics 24″ for participants to be able to adjust the screen to their eye level if needed. Each stimulus was presented using the experimental control software PsychoPy3. In each trial, participants typed their response directly on the display using the Roman alphabet keyboard (as Japanese orthography has no adequate way of encoding coda consonants). Participants’ responses and response times for each trial were logged by PsychoPy3. At the end of the experimental session, the primary investigator informally asked each participant if there was anything they noticed during the experiment.

### Data Analysis

Each participant’s typed responses were transcribed before the actual data analysis. Since Japanese orthography does not have a spelling convention to express word-final consonant, the Roman alphabet was used for both language groups to report what they heard. Because all of the Japanese speakers had some exposure to alphabetic writing systems, they did not report significant difficulty with this request, and their answers generally adhered to the forms of English orthography (e.g., participants did not provide ill-formed sequences like “ihpk”). As we will see in the audio-visual congruent results, the Japanese speakers performed near-ceiling when the data were sufficiently clear, suggesting that responding in English orthography did not present a significant difficulty for the Japanese-speaking participants.

As English orthography sometimes maps multiple letters onto a single sound, some responses required translation into a phonemic form (e.g., word-initial “c” and “ch” as representations of the sound [k]). Generally, these spelling conventions were straightforward, and we developed a set of shared rules for both languages’ participants to convert the data from letter strings to sounds (e.g., word-initial “c,” “ch,” “ck,” and “c” transcribed as /k/; see Appendix A for the full spelling conversions). After this conversion, the reported sounds were checked against the true values of the stimuli. Each response was categorized into one of the six categories listed below. Some categories are restricted to certain conditions, which are listed in brackets (e.g., an audio-only recording cannot be in category V, as it cannot be consistent with non-existent video):

• Audio response (A): response that shares place and manner of articulation features of the audio component [audio-only or audio-visual incongruent].
• Visual response (V): response that shares place and manner of articulation features of the video component [audio-visual incongruent only].
• Audio-visual response (AV): response that shares place and manner of articulation features of both audio and video components [audio-visual congruent only].
• No consonant response (NA): response lacks a consonant in the onset or coda position being tested [all conditions].
• Mid-fusion response (midF): response of a single consonant that reflects the perception of a third sound which is different from both audio and visual information but at an articulatory place between them [audio-visual incongruent only].
• Two-letter fusion response (bothF): response of multiple consonants with one consistent with audio and another consistent with visual information when stimulus is audio and another consistent with visual information when stimulus is audio-visually incongruent [audio-visual incongruent only].
• Other response (O): response that had at least one consonant in the target position but does not fit any other above category [all conditions].

Some research treats audio-visual fusion effects, where the perceived sound lies articulatorily between the audio and video phones, as the only relevant McGurk effect, while others include any deviation from the audio information as a McGurk effect (Tiippana, 2014). In the present study, in order to make out data comparable to those from the study of Ali et al., only the consonants that represent the articulatory midpoint of auditorily and visually presented phones are considered to be McGurk fusion. Voicing values were disregarded (e.g., “b” and “p” were treated as equivalent), since all target sounds were voiceless, and the intended McGurk effects only affected the perceived place of articulation. Lastly, participants occasionally included multiple consonants in the target positions. When these agreed in place and manner, or when one was a stop and the other was not, they were treated as a single stop and classified as above. When they formed a common English digraph (e.g., “ch,” “sh”), they were also treated as a single sound. Other cases are listed in Appendix A.

## Results

We will discuss each of the three conditions (audio-only, audio-visual congruent, and audio-visual incongruent) in order. The audio-only data will establish the baseline confusability of the auditory components for audio-visual cases, in addition to directly testing the Phonetic- and Phonological-Superiority Hypotheses. The audio-visual congruent case will establish how participants are able to incorporate additional cues, providing a sense of how much they rely on each modality. Finally, considering the audio-visual incongruent case will test potential Phonological-Superiority effects.

### Audio-Only Block

Table 3 summarizes the participants’ overall percentage of correct responses for the target consonants, split by consonantal position, in the audio-only stimuli. Binomial tests showed that English speakers perceived both onset and coda target consonants in monosyllabic CVC words significantly above chance (onsets: *p* < 0.001, codas: *p* < 0.05), as expected. Japanese speakers perceived the target consonants significantly above chance when they appear in onset position (*p* < 0.001), but perceived them significantly *below chance* in coda position (*p* < 0.01). Furthermore, both language groups showed significant differences in onset and coda accuracy, with both groups having lower accuracy on codas (English speakers: χ^2^ = 6.1295, df = 1, *p* < 0.05; Japanese speakers: χ^2^ = 25.115, df = 1, *p* < 0.001). Thus, overall accuracy patterns in identifying the target consonants [p], [t], and [k] were similar across the two language groups, with lower overall accuracy in Japanese, and in coda positions. This seems to be consistent with a primarily phonetic influence on perception, since English speakers have no phonotactic reason to underperform on coda identification.

**TABLE 3:** Audio-only accuracies for onsets and codas in CVC stimuli.

	English CVC		Japanese CVC	
	Onset	Coda	Onset	Coda
Correct response (%)	87.0***	64.8*	77.8**	27.8*

These results aggregate [p], [t], and [k] target consonants. All performances are significantly greater than 50% by a binomial test, with the exception of Japanese coda perception, which is significantly *below* 50% (*p < 0.05; **p < 0.01; and ***p < 0.001).

The phonetic influence on perception accuracy suggests that different phones have different baseline accuracies, either due to differences in how easy it is to distinguish the phones, or due to differences in the quality of the individual recordings of the phones in our experiment. Further analysis split the above results based on the specific target consonants, as shown in Table 4. This analysis found that some consonants had significantly different accuracies, which depended on consonant identity, onset/coda position, and language.

**TABLE 4:** Audio-only accuracies for onsets and codas in CVC stimuli, split by correct consonant identity.

	English listeners					
	Onset			Coda		
	/p/	/t/	/k/	/p/	/t/	/k/
Correct response (%)	72.2	88.9**	100***	61.1	50.0	83.3**

	**Japanese listeners**					
	**Onset**			**Coda**		
	**/p/**	**/t/**	**/k/**	**/p/**	**/t/**	**/k/**

Correct response (%)	44.4	94.4***	94.4***	22.2*	16.7**	44.4

Significance marked when accuracy greater than chance by a binomial test, with the exception of Japanese coda perception, which is significantly *below* 50% (*p < 0.05; **p < 0.01; and ***p < 0.001).

Binomial tests show that the English speakers’ accuracy in perceiving onset [t] and [k] were significantly higher than chance (*p* < 0.01), while the difference was not significant in onset [p] perception. For English coda perception, [k] was perceived significantly more accurately than chance (*p* < 0.01), but not [p] or [t]. Japanese speakers’ response patterns for onset target consonants were similar to those of English speakers, with significantly greater than chance performance on [t] and [k], but not [p]. In coda position, no consonant was perceived above chance by the Japanese speakers. Numerically, however, the same basic pattern appears in both languages, in both positions, with the exception of Japanese coda [k] perception. Again, there is no obvious evidence for large-scale phonotactic influences on accuracy across the two languages, and instead, individual phonetic differences seem to be the best explanation for this pattern.

However, binomial statistical tests compare the accuracy against chance, rather than against each other. The Phonological-Superiority Hypothesis predicts that speakers of Japanese will be relatively more affected by coda-position difficulties than English speakers are. We can directly test this, by using a logistic regression model, fit by R’s *glm* function, that predicts participant accuracy based on three control factors: participant’s L1, consonant position, and consonant identity. We also include two interaction terms: the interaction between L1 and consonant position, and between L1 and consonant identity. The first interaction is where we expect to see an effect if there is a phonotactic influence on the accuracies; the second is included as a control in case some of the consonants are easier or harder to identify based on L1 phonology. Table 5 contains the results of this model.

**TABLE 5:** Logistic regression coefficients (log-odds change, with standard error in parentheses) for the audio-only model.

Intercept (English, onset, [k])	Japanese L1	Position is coda	[p]	[t]	Japanese codas
3.29 (0.73)***	−0.81 (0.93)	−1.39 (0.51)**	−1.81 (0.72)*	−1.67 (0.72)*	−1.21 (0.74)

The intercept is estimated accuracy for an English-speaking participant identifying a [k] in onset position (*p < 0.05; **p < 0.01; ***p < 0.001).

The values in Table 5 are the log-odds effects of a change to the “default” feature of an English-speaking participant identifying a [k] in onset position, the situation with the best performance. Based on these values, for instance, an English speaker identifying a [t] in the onset position would have log-odds of a correct identification of 1.62 (the default 3.29, minus 1.67 for switching from [k] to [t]). Interpreting log-odds is slightly complex, but the key factors identified here are that switching from onset to coda has a significant negative effect on accuracy, as does switching between the individual phones. However, the effect of Japanese as an L1 instead of English is numerically negative, but not significant. Likewise, our key factor for identifying a phonological effect—the interaction between L1 and consonant position—is also not quite significant (*p* = 0.10). (The L1-consonant interaction was also insignificant, and omitted from the table).

Overall, it seems that there may be a small phonotactic/phonological effect in the audio-only data, but strongest, and only significant, effects appear to be more consistent with phonetic influences. [k] appears to be an especially identifiable consonant, compared to [p] and [t], and onsets appear to be more recognizable regardless of a listener’s preferred phonotactics. However, the large numeric drop-off in Japanese coda performance suggests that phonological knowledge plays an important role as well. We will continue this analysis with the audio-visual congruent data below.

### Audio-Visual Block

#### Audio-Visual Congruent

The McGurk effects that Ali et al. (2011) tested rely on an audio-video mismatch. To establish a baseline performance, we first look at audio-visual congruent data, where the participant sees the actual video of the speaker pronouncing the words. The perception accuracies for each target consonant in different positions are provided in Table 6, and they are almost uniformly above chance.

**TABLE 6:** Audio-visual congruent accuracies for onsets and codas in CVC stimuli, split by correct consonant identity.

	Congruent					
	English listeners					
	Onset			Coda		
	AV[pp]	AV[tt]	AV[kk]	AV[pp]	AV[tt]	AV[kk]
Correct response (%)	94.4***	100***	100***	94.4***	94.4***	100***

	**Japanese listeners**					
	**Onset**			**Coda**		
	**AV[pp]**	**AV[tt]**	**AV[kk]**	**AV[pp]**	**AV[tt]**	**AV[kk]**

Correct response (%)	94.4***	100***	94.4***	94.4***	66.7	83.3***

Significance marked when accuracy greater than chance by a binomial test (***p < 0.001).

Participants benefited greatly from the visual information, with uniformly higher scores than the audio-only condition. In aggregate, this is unsurprising, since the audio-only stimuli were embedded in multi-talker babble that introduced significant noise. The relatively clean visual data were especially helpful for identifying the lip closure that differentiates [p] from the non-labial [t] and [k] sounds, but surprisingly, visual information was also extremely helpful in distinguishing between [t] and [k] sounds, despite them looking very visually similar. Visual information also appears to have been effectively used by speakers of both languages, with the only accuracy that was not significantly greater than chance being Japanese [t] codas, which had the lowest accuracy in the audio-only condition. Even still, visual information had the single greatest impact for Japanese coda identification, and Japanese [t] coda accuracy was significantly higher in audio-visual than audio-only by a chi-squared test (χ^2^ = 7.3143, df = 1, *p* < 0.01). All the consonants that were not perceived accurately in the audio-only condition were perceived correctly when compatible visual information was provided. This confirms that listeners, especially when the audio input is noisy, rely on visual information in order to perceive and process speech cross-linguistically.

These results are more in line with the Phonetic-Superiority Hypothesis. The only conditions that are not essentially at ceiling are Japanese listeners identifying coda consonants. The visual information seems to be sufficient to overcome the phonetic noise, and the remaining difficulty is focused on the one phonotactically illicit position. This suggests that both the Phonetic- and Phonological-Superiority Hypotheses may be slightly off target. Instead, it appears that different tasks can induce stronger phonetic or phonological effects. Furthermore, the ability of both language groups to marshal visual information when the audio information is weak suggests that phonetic and phonological information is only one of the informational components used in phonemic identification. This suggests a more complex alternative hypothesis, that phonetic and phonological factors are integrated in an information-based framework, based on the perceived reliability of each cue type in the present task. We will discuss this possibility further in the section “Discussion,” but first, let us turn to the audio-visual incongruent data to examine McGurk fusion effects across the languages.

#### Audio-Visual Incongruent

Given how much of a factor visual information played in the AV-congruent data, we expect to see significant McGurk effects in the AV-incongruent data. We categorize the responses to understand how exactly participants resolved the conflicts, focusing on four critical categories of responses in these data, based on the division in section “Data Analysis”:

• A: an answer consistent with the audio, rather than visual, information.
• V: an answer consistent with the visual, rather than audio, information.
• F: an answer that fuses the two modalities by producing a place of articulation between the audio and visual components (e.g., reporting [t] when hearing [p] but seeing [k]).
• O: all other answers.

The more fine-grained distinction will help reveal the relative importance and confidence that participants assigned to each of the modalities. An A or V response reflects higher confidence in audio or visual information, respectively. An F response reflects comparable importance assigned to both modalities, leading the participant to seek middle ground that is partially consistent with each. An O response reflects low confidence in both, leading participants to choose options that are inconsistent with both modalities. Overall, we will see that participants change their responses in line with this trade-off between confidence levels in the information that each modality supplies, suggesting the need for a more nuanced integration of phonetic, phonological, and other sources of information in L2 phonetic categorization than the superiority hypotheses offer.

We analyzed two types of audio-visual incongruent pairs separately: (a) fusible incongruent stimuli and (b) non-fusible incongruent stimuli. Fusible incongruent stimuli consisted of an audio /p/ and visual /k/ (AV[pk]) or audio /k/ and visual /p/ (AV[kp]). McGurk fusion on these cases results in perception of /t/ (midF response category), an articulatory midpoint between the two inputs. The non-fusible incongruent stimuli are ones composed of audio /p/ or /k/ with visual /t/ (AV[pt] and AV[kt]). There is no articulatory midpoint between the inputs, so “fusion” responses are not possible (see Table 7).

**TABLE 7:** Proportion of responses in each category for fusible audio-visual incongruent stimuli.

	Incongruent			
	English speakers			
	Onset		Coda	
	AV[pk]	AV[kp]	AV[pk]	AV[kp]
A	11.1	**83.3**	–	5.6
V	11.1	16.7	**55.6**	**77.8**
F	**77.8**	–	5.6	–
O	–	–	38.9	16.7

	**Japanese speakers**			
	**Onset**		**Coda**	
	**AV[pk]**	**AV[kp]**	**AV[pk]**	**AV[kp]**

A	5.6	**94.4**	5.6	11.1
V	–	5.6	33.3	**66.7**
F	**94.4**	–	11.1	11.1
O	–	–	**50.0**	11.1

*Dashes indicate no response in that category for that stimulus. Most frequent responses for each L1/stimulus combination are bolded.*

Beginning with the fusible results, shown in Table 7, the most obvious pattern is that the choice of audio and video data has a significant effect on participants’ preferences. In onset positions, for both Japanese and English listeners, audio [p] with visual [k] (the AV[pk] condition) induced primarily fusion responses of [t]. Listeners appear to have similar trust in the audio and visual datastreams, and seek out a compromise response that does not favor one over the other. This was significantly different, by a chi-squared test (χ^2^ = 30.066, df = 1, *p* < 0.001), from onsets with audio [k] and visual [p] (the AV[kp] condition) for each L1. Audio [k] in onset was favored by listeners from both L1s, with no fusion responses, suggesting that this audio information was viewed as more reliable than the video information.

The audio-only results are helpful in understanding this switch. In onset positions, listeners from both L1s found [p] harder to identify than [k]. When the audio was less reliable ([p]), listeners incorporated the visual data to help their categorization, leading to fusion. When the audio was more reliable ([k]), the visual data were overruled by the audio. It appears that listeners, regardless of language, reach their categorization decisions by balancing the information that each modality provides against the perceived reliability of that cue’s information.

A similar pattern emerges for codas. In the AV[pk] condition, both English and Japanese listeners respond primarily with visually consistent or other responses (critically, they only rarely respond with audio-consistent or fusion responses). Again, this reflects the participants’ perception of the cue reliability; coda [p] had very low accuracy in the audio-only condition in both languages, so non-audio information dominates. The AV[kp] condition may be a little surprising; coda [k] accuracy was high for English listeners, yet both English and Japanese listeners favor visual information in the incongruent presentation. However, visual [p] with lip closure is stronger visual information than visual [t] or [k], and this may explain the phenomenon.

Both English and Japanese speakers fused significantly more when auditorily visually incongruent AV[pk] was presented than AV[kp] (χ^2^ = 30.066, df = 1, *p* < 0.001). In AV[pk] perception, fusion was significantly more common in onset position than coda position. In AV[kp] perception, speakers of both languages preferred auditorily consistent answers in onset position whereas they showed a strong preference for visually consistent answers in coda position. Note that McGurk fusion is quite rare in this dataset, and only occurs in appreciable amounts in onset positions with [p] audio.

Non-fusible stimuli showed a similar overall pattern to the fusible stimuli. Visual [t] dominated auditory [p] regardless of language background and syllabic position, although in coda position, many responses were inconsistent with either the auditory or visual information. On the other hand, auditory [k] dominated visual [t] regardless of language background or syllabic position. Again, this appears to be consistent with participants preferring the input cue that they find more reliable, rather than relying on phonetic or phonological information across the board.

To test the idea that the perceived auditory reliability influences how closely participants adhere to the auditory and visual inputs, we performed a post-hoc correlation test. For each of the incongruent stimuli, we used the audio-only accuracies from Table 4 to predict the rate of audio-consistent responses in Tables 7, 8. The basic idea is that when participants are able to use the audio information to categorize the sound, they prefer to rely on it. When the audio information is noisy or otherwise unreliable, they turn to whatever other information is available, whether that is visual information, phonological preferences, or something else. If none is perceived as reliable, they guess.

**TABLE 8:** Proportion of responses in each category for non-fusible audio-visual incongruent stimuli.

	Incongruent			
	English listeners			
	Onset		Coda	
	AV[pt]	AV[kt]	AV[pt]	AV[kt]
A	5.6	**100**	–	**100**
V	**94.4**	–	44.4	–
O	–	–	**55.6**	–

	**Japanese listeners**			
	**Onset**		**Coda**	
	**AV[pt]**	**AV[kt]**	**AV[pt]**	**AV[kt]**

A	11.1	**88.9**	–	**72.2**
V	**88.9**	11.1	16.7	5.6
O	–	–	**83.3**	22.2

*Dashes indicate no response in that category for that stimulus. Most frequent responses for each L1/stimulus combination are bolded.*

**FIGURE 1:**
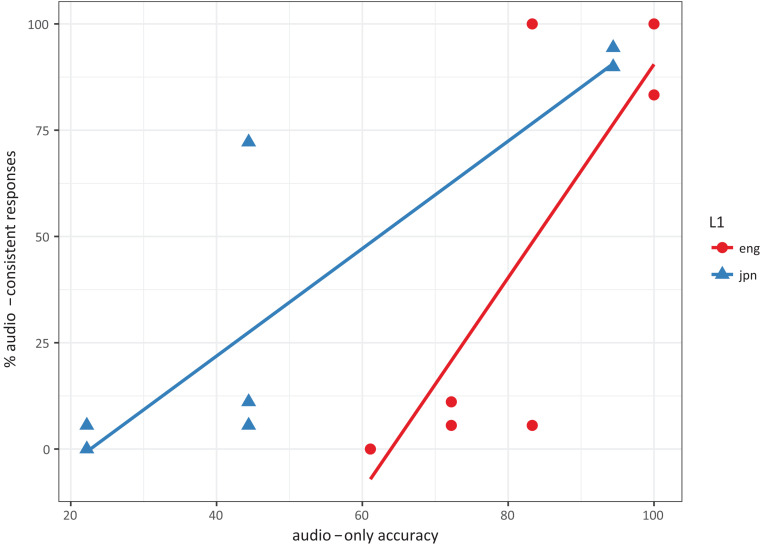
Scatterplot of audio-only accuracy and audio-consistent responses across L1, syllable position, and consonants. Correlations were significant for both languages.

The correlation pattern is shown in Figure 1. Each dot represents an L1, syllabic position, and consonant identity combination. Correlations were fit separately for each language, to account for individual preferences toward one modality over the other; for instance, Hayashi and Sekiyama (1998) found different preferences for visual information cross-linguistically. In both languages, the correlation test found a significant correlation between audio-only accuracy and audio-consistent responses (Japanese: *t* = 4.18, df = 6, *p* < 0.01; English: *t* = 3.51, df = 6, *p* < 0.05), which fits with the idea that participants adjust their answers to account for the reliability of the auditory information they are receiving.

Overall, unlike the results from previous studies (e.g., Ali et al., 2011), the results from the audio-visual incongruent condition show more fusion in the onsets than in the codas, and do not show an interaction of consonant position and listeners’ L1 phonology. This seems to be an argument against the Phonological-Superiority Hypothesis. However, participants’ reliance on auditory information is significantly positively correlated with the audio-only accuracy, and patterns slightly differently in each language.

## Discussion

The current study was conducted to further assess the influence of L1 phonology on non-native speech perception and the McGurk fusion effect, beyond the findings of Ali et al. (2011). To focus on syllable structural influence on speech perception, in addition to English, which is open to CVC syllables, Japanese was tested to conduct the baseline as the language predominantly prefers CV syllables but has a strict restriction on consonants in coda position. Moreover, in order to investigate influence of acoustic quality and listeners’ phonological knowledge (e.g., phonotactic constraints), instead of real words, we created monosyllabic nonsense words composed of phonemes that are common in the English and Japanese phonemic inventory.

We first conducted audio-only condition to further investigate the influence of the stimuli’s acoustic quality and listeners’ L1 phonotactic knowledge over the course of speech perception. We used this condition to look for phonetic and phonological influences, testing the Phonetic- and Phonological-Superiority Hypotheses. There was clear evidence of phonetic influences, depending on the syllable position and phoneme identities. Phonological influence, in the form of a difference between Japanese and English listeners’ performance on onset versus coda consonant identification, was numerically present, but did not reach significance. Within this experiment, the Phonological-Superiority Hypothesis appears to be invalid, but the Phonetic-Superiority Hypothesis may not hold either.

The overall results from the audio-only condition demonstrated that both the acoustic quality of the audio input and the listener’s phonological knowledge can influence perception of unfamiliar speech sounds, with clearer evidence for an effect of acoustic quality. Although Japanese listeners reported lower accuracy for coda consonant perception than English listeners, the fact that English listeners did not perceive all the coda consonants accurately suggests that listeners combine both acoustic and phonological information available in input and in their mental representation over the course of speech perception, especially when the input data are noisy.

To probe the nature of this relationship more carefully, we introduced visual information that was either consistent with the audio information or inconsistent with it. When the visual information was consistent with the audio information, participants were able to achieve high rates of phonemic categorization accuracy, even for consonants that were very difficult in the audio-only condition. This indicates that people “listen” to more than just acoustic signals available in input and integrate multiple cues in order to extract more precise estimates of the phonemic identity, even in unfamiliar languages. The relatively higher accuracy for consonant perception in both syllable positions by both language groups in the audio-visual congruent condition suggests that visual information provides strong information, and is especially important when the auditory information is incomplete. With sufficiently clear L2 input (i.e., auditorily and visually presented information), phonetic and phonological effects on phonemic categorization can be overcome—at least when the phonemic categories are familiar from the L1.

Taking these observations into consideration, we argue that neither Phonetic- nor Phonological-Superiority Hypotheses can fully explain the nonnative speech perception. Rather, when listeners perceive speech sounds in natural setting, they combine multiple cues such as acoustic information, phonological knowledge of the language(s) previously acquired, and visually presented information regarding articulation altogether in order to resolve the difficulty identifying unfamiliar sounds. In some situations, this can manifest as a Phonetic-Superiority Effect (e.g., when the phonetic information is strong enough to minimize phonological difficulties). In others, this can manifest as Phonological-Superiority (e.g., when phonetic information is comparable in different syllabic positions). In still other situations, other factors can dominate, such as the visual information in some of the congruent and incongruent conditions.

So far, these results show that pure speech perception is influenced by multiple information types available both in the input and in the listener’s mental representation. Indeed, it is a well-known fact that listeners attend to multiple cues to different extent depending on the tasks and quality of input during the course of non-native speech perception (Detey and Nespoulous, 2008; Escudero and Wanrooij, 2010). What is interesting though is that listeners seem to combine not only auditorily and visually presented cues but also their L1 and/or L2 knowledge even in the situation where they are provided with inconsistent inputs from different modalities.

When the inputs conflict, how do learners resolve the conflicts? Unlike the findings reported in previous studies (e.g., Ali et al., 2011), in the audio-visual incongruent condition in the present study, both English and Japanese speakers showed very similar McGurk effects, including in the particular case of McGurk fusion, in each consonant position. The only stimulus that elicited a strong McGurk fusion effect was AV[pk] (audio /p/ with visual /k/) in onset position but not in coda position. This is the opposite of what was originally found among English speakers in the previous studies, which found increased fusion in codas. Instead, we found elevated rates of responses that were not consistent with the audio information, but were not necessarily fusion responses.

There are potentially three explanations for the difference in the results reported in the previous studies from the results demonstrated in the current study. The first explanation is that, in the previous studies, additional information other than acoustic or phonological (e.g., semantic information) may have been available in stimuli since researchers used real words in English and Arabic. As a result, their English listeners’ perception in Arabic stimuli may have been influenced by other than simple phonetic- or phonologically driven factor but something else, such as lexical knowledge or frequency of the word. Consequently, the previous results showed a different fusion pattern because of possible factors influencing speech perception other than phonetic- or phonological-information. The second possible explanation for Japanese speakers in the current study showing the fusion patterns similar to English listeners is that Japanese listeners’ exposure to English may have changed the way Japanese participants perceiving non-native sounds. Although most of our Japanese participants were native Japanese speakers and had never lived in a country where English is spoken as a common language for more than a year prior to the research participation, almost all of them were students studying English at ALI (American Language Institute) in San Diego. Though most of them were tested within 30 days after their arrival to the U.S., the exposure to English may have made them more familiar with coda consonants. Thus, it is reasonable to argue that although Japanese phonology does not allow to have /p/, /t/, and /k/ in coda position unless it is geminated, the Japanese participants in the present study perceived stimuli in a similar way to English speakers. Consequently, there was no significant cross-language difference in McGurk fusion rates between current English-speaking participants and Japanese-speaking participants. Lastly, the difference between the results from the present study and previous studies may be reflective of a phonetic influence from the specific stimuli being used. Audio stimuli used in the present study was less reliable due to the added noise, compared to those used in previous studies. Since visual information was essentially noiseless compared to the audio files, our participants may have been more reliant on visual information.

## Conclusion

The present study suggests a strong evidence of listeners integrating multiple cues available in input such as acoustic information and visual information as well as knowledge they had built based on previous language learning experience and phonological knowledge of language or languages previously acquired. Although how and when they shift their reliance on each cue is unanswered, the current results from three separate conditions suggest that listeners integrate acoustically and visually presented information available in input as well as phonological knowledge over the course of speech perception. Also, learners unconsciously balance their reliance on different information during the course of speech perception depending on the certainty they established regarding each type of information, and the reliability of each cue available in input.

In order to further investigate how listeners would integrate acoustic-, visual-, and phonological-information cues available in input when audio and visual cues disagree in terms of the place of articulation, a separate data from visual-only condition are required. Is visual information more dominant than audio information or vice versa? Or do listeners attempt to resolve this disagreement across two cues presented at the same time in a way that is consistent with both? A complete answer to these questions will require further research. However, the findings from the present study provide insights into the bilingual and multilingual speech perception process and influences of L1 and L2 structure when they encounter speech sounds in an unfamiliar language.

## Data Availability Statement

The datasets generated for this study are available on request to the corresponding author.

## Ethics Statement

The studies involving human participants were reviewed and approved by Graduate and Research Affairs, San Diego State University. The participants provided their written informed consent to participate in this study.

## Author Contributions

KY and GD contributed to conception and design of the study, performed the data analysis, contributed to manuscript revision, and read and approved the submitted version. KY collected data. KY wrote the first draft of the manuscript. Both authors contributed to the article and approved the submitted version.

## Conflict of Interest

The authors declare that the research was conducted in the absence of any commercial or financial relationships that could be construed as a potential conflict of interest.

## Appendix A

1. “c” and “ch” in word initial position are mostly transcribed as /k/ (this is because both “c” and “ch” responses were common in “k” stimuli but virtually unattested for “t” stimuli) except when the response started with “cer-” or “cea-,” in which case /s/ was used, since the letter “c” in those contexts is usually pronounced as [s] in English.
2. “ck” and “c” occurring word-finally were transcribed as /k/ (since in English word-final “ck” and “c” are usually recognized as /k/, as in “dock” or “tactic”).
3. “gh” or “ght” at the end of the word were transcribed as /w/ or /f/.
4. Homorganic consonant clusters (a sequence of two or more consonants that have the same place of articulation, like [dt]0 appearing in word-medial positions) were coded as a combination of a glottal stop followed by the consonant (since, in Japanese, a word-medial homorganic consonant cluster is usually recognized as a combination of a glottal stop followed by the consonant, as in “katto” pronounced as [kaʔto]).

Learning to move from auditory signals to phonemic categories is a crucial component of first, second, and multilingual language acquisition. Yet, especially in the case of second and/or later language acquisition, learners are confronted with difficulties to accurately perceive unfamiliar sounds. This difficulty may be induced due to the acoustic informativity (Phonetic-Superiority Hypothesis) or interference of listeners’ phonological knowledge that they have built based on the previous language exposure (Phonological-Superiority Hypothesis). The present study carefully tested the influence of acoustic informativity and learner’s phonological preferences during speech perception. The findings from the present study suggest that listeners integrate multiple cues available (acoustic, visual, and phonological cue) during the course of speech perception. Based on the current findings, we propose a cognitively inspired rational cue integration framework as a third hypothesis to explain how L1 phonological knowledge affects L2 perception. The findings from the current study provide insights into the bilingual and multilingual speech perception process and influences of L1 and L2 structure when they encounter speech sounds in an unfamiliar language.
